# *Stenotrophomonas maltophilia* bacteremia in adult patients with hematological diseases: clinical characteristics and risk factors for 28-day mortality

**DOI:** 10.1128/spectrum.01011-24

**Published:** 2024-11-29

**Authors:** Wenjing Guo, Qingsong Lin, Jia Li, Xiaomeng Feng, Sisi Zhen, Yingchang Mi, Yizhou Zheng, Fengkui Zhang, Zhijian Xiao, Erlie Jiang, Mingzhe Han, Jianxiang Wang, Sizhou Feng

**Affiliations:** 1 State Key Laboratory of Experimental Hematology, National Clinical Research Center for Blood Diseases, Haihe Laboratory of Cell Ecosystem, Institute of Hematology & Blood Diseases Hospital, Chinese Academy of Medical Sciences & Peking Union Medical College, Tianjin, China; 2 Tianjin Institutes of Health Science, Tianjin, China; UJF-Grenoble 1, CHU Grenoble, Grenoble, France

**Keywords:** hematological disease, *Stenotrophomonas maltophilia*, bacteremia, risk factors, treatment

## Abstract

Patients with hematological diseases are at high risk for *Stenotrophomonas maltophilia* (SM) bacteremia. This study retrospectively analyzed the clinical characteristics and risk factors for 28-day mortality among 140 adult hematological patients diagnosed with SM bacteremia from January 2012 to July 2023. he overall 28-day mortality was 31.43% (44/140), with a median age of 44 years. The median hospital stay before SM bacteremia onset was 25 days, and 69.29% of patients had unresolved neutropenia. All patients had received broad-spectrum antibiotics in the past month, and 69.29% developed breakthrough bacteremia during carbapenem therapy. Independent risk factors for mortality included a Sequential Organ Failure Assessment (SOFA) score ≥5, tigecycline exposure, age ≥60, and pulmonary infection. Patients with ≥2 risk factors were stratified into the high-risk group, with a significantly higher 28-day mortality compared with the low-risk group (56.52% vs 7.04%, *P* < 0.001). Treatment with trimethoprim-sulfamethoxazole (TMP/SMX) (*P* = 0.008) or TMP/SMX combined with cefoperazone/sulbactam (CSL) (*P* = 0.005) was associated with survival benefits among high-risk patients. Overall, SM bacteremia usually occurs in hematological patients with prolonged hospitalization, unresolved neutropenia, and extensive use of broad-spectrum antibiotics, especially carbapenems. Patients with high SOFA scores, advanced age, pulmonary infection, or recent tigecycline exposure are at higher risk of mortality. The preferred treatment is TMP/SMX rather than fluoroquinolones, with combination therapy of TMP/SMX and CSL considered a feasible treatment option.

**IMPORTANCE:**

This study, representing the largest cohort of adult hematological patients with SM bacteremia to date, strengthens the validity of existing findings and provides new insights into its clinical management. We identify risk factors for 28-day mortality, revealing that patients with two or more risk factors experience particularly high mortality rates. This highlights the importance of early identification and targeted management of high-risk individuals. Our findings also demonstrate that TMP/SMX is superior to fluoroquinolones and suggest that combining TMP/SMX with CSL may offer additional survival benefits.

## INTRODUCTION

*Stenotrophomonas maltophilia* (SM) is a Gram-negative non-fermentative bacterium capable of adhering to respiratory epithelial cells and invasive medical devices. This adherence facilitates biofilm formation, enhancing its colonization and resistance to host defense (1). SM harbors numerous antibiotic resistance genes and produces L1 metallo-β-lactamase and L2 serine β-lactamase, which confer intrinsic resistance to most β-lactam antibiotics, including carbapenems and cephalosporins (2). Additionally, SM can develop resistance to aminoglycosides and fluoroquinolones through various enzymatic mechanisms and efflux pumps (1). Consequently, although SM is not a major pathogen in bacteremia during initial fever or neutropenia, it frequently causes bacteremia in patients with prolonged neutropenia (3) or those receiving broad-spectrum antibiotics for existing infections (4–6).

Mortality rates associated with SM bacteremia in the general population vary between 16.8% and 54.8% (7–14). Hematological patients, characterized by extended hospital stays, central venous catheter use, compromised immune systems, and altered microbiomes, are particularly vulnerable to SM infections (15). Consequently, they often experience more severe clinical courses and poorer outcomes (8, 14), with crude mortality rates for SM bacteremia ranging from approximately 21.9% to 67.3% (7, 12, 16–20).

However, existing studies on SM bacteremia in hematological patients are limited, with relatively small sample sizes, and the optimal therapy remains undetermined. In this retrospective analysis, our objective was to investigate the clinical features and identify mortality risk factors in a larger cohort of adult hematological patients with SM bacteremia. Our findings may help identify high-risk patients and provide treatment recommendations to improve clinical outcomes.

## MATERIALS AND METHODS

### Study setting and population

This retrospective analysis of 140 patients was performed in a 1,540-bed tertiary blood disease center in Tianjin, China. All patients were ≥14 years old and tested positive for SM in one or more blood cultures from January 2012 to July 2023. For patients who had more than one episode of SM bacteremia, only the first one was collected. Patients with incomplete medical records were excluded (*n* = 3), and 140 patients with SM bacteremia were enrolled in this study. This study was approved by the Ethics Committee of the Institute of Hematology and Blood Diseases Hospital, Chinese Academy of Medical Sciences.

### Data collection

The following information was collected from electronic medical records: age, sex, weight loss in preceding 1 month, length of hospital stay before SM bacteremia, central venous catheter, temperature, Sequential Organ Failure Assessment (SOFA) score, co-infections, underlying hematological diseases and related treatment, antibiotic therapy regimen, previous SM infection at other sites, polymicrobial bacteremia, neutropenia, source of infection, survival status at 28 days after the onset of bacteremia, and *in vitro* susceptibility to antibiotics.

### Definitions

SM bacteremia was defined as the presence of one or more positive blood cultures for SM in a patient with clinical symptoms. The onset of SM bacteremia was defined as the date of collection of the positive blood culture sample. Polymicrobial bacteremia was defined as two or more bacterial species identified in multiple blood culture samples collected within 72 h. Neutropenia was defined as peripheral blood absolute neutrophil count (ANC) < 500/mm^3^. Febrile neutropenia was defined as oral temperature ≥38.0°C over an hour or ≥38.3°C once while experiencing neutropenia (21). The source of bacteremia was characterized by the isolation of SM in specimens obtained from other sites, including sputum, oropharynx, perianal area, skin, and soft tissue, along with concurrent localized active infection symptoms. Otherwise, it was classified as a primary infection (12). To facilitate the timely detection of infection sources, patients underwent sputum cultures and were routinely tested for bacterial colonization in the nasal cavity, pharynx, perianal skin, and catheter sites using cotton swabs dipped in 0.9% sterile saline twice a week. Glucocorticoid use was defined as a daily dose of ≥10 mg prednisolone-equivalent steroid within 1 month before bacteremia onset. The intravenous antibiotic or oral trimethoprim-sulfamethoxazole (TMP/SMX) administration for at least 48 h within 1 month before bacteremia onset was regarded as prior antibiotic use. Appropriate empirical therapy was defined as the administration of 1 or more agents with *in vitro* activity no later than 48 h after the first blood sample collection. Monotherapy was defined as treatment with only one active antimicrobial agent, and combination therapy was defined as treatment with >1 active antimicrobial agent. Overall mortality was defined as death from any cause occurring within 28 days of the onset of SM bacteremia.

### Microbiology

Antibiotic susceptibility testing was performed in the hospital’s microbiology laboratory using an automated system (VITEK 2 Compact) with broth microdilution and disk diffusion methods. The interpretation of antibiotic susceptibilities followed the guidelines provided by the Clinical and Laboratory Standards Institute (CLSI) M100 standard (22) and the European Committee on Antimicrobial Susceptibility Testing (EUCAST) (23). For agents whose specific CLSI and EUCAST criteria for SM were not available, we utilized the relevant criteria for *non-Enterobacteriaceae* (16).

### Statistical analysis

The 140 patients were divided into two groups based on their survival status 28 days after the first positive culture was obtained. Continuous variables are presented as mean (standard deviation, SD) or median (interquartile range, IQR) and compared using the Student’s *t*-test or the Mann–Whitney *U* test. Categorical data were summarized as counts (percentage, %) and compared using the χ^2^ test or Fisher’s exact test. All potentially meaningful variables with a univariate *P* value < 0.1 were enrolled in a multivariate logistic regression model using stepwise selection to identify risk factors for 28-day mortality in SM bacteremia. The results were expressed as odds ratio (OR) with 95% CI (CI). Continuous variables were categorized before inclusion based on clinical significance or the optimal cutoff value of the receiver operating characteristic curve (ROC). Multicollinearity was measured via the variance inflation factor (VIF), with values < 2 considered acceptable. Patient survival was evaluated using Kaplan-Meier curves and log-rank tests.

All statistical analyses were performed with RStudio (version 4.3.1). Statistical significance was set at a two-sided *P* value < 0.05.

## RESULTS

### Patient characteristics

The overall mortality rate among 140 adult hematological patients with SM bacteremia was 31.43%. Patients were categorized based on their 28-day survival status, and their clinical characteristics were compared (Table 1). The median age was 44 years (IQR: 30.00–55.25), with 22 patients (15.71%) aged over 60 years, and 51 patients (36.43%) were female. A trend toward higher mortality was observed in patients aged 60 years or older (*P* = 0.073). The predominant underlying diseases were AML (58 cases), AA (35 cases), and ALL (24 cases).

**TABLE 1:** Clinical features of adult hematological patients with SM bacteremia. Non-survivors and survivors were compared*^a^*^,^*^b^*

	Total *n* = 140	Non-survivor *n* = 44	Survivor *n* = 96	*P* value
Age, years (median, [IQR])	44.00 [30.00, 55.25]	46.50[37.75, 59.25]	42.00 [28.00, 54.25]	***0.051***
Age ≥ 60 years	22 (15.71)	11 (25.00)	11 (11.46)	***0.073***
Female sex	51 (36.43)	18 (40.91)	33 (33.38)	0.578
Weight loss ≥5 kg in preceding 1 month	47 (33.57)	16 (36.36)	31 (32.29)	0.779
Length of hospital stay before SM bacteremia, days	25.00 [18.00, 48.50]	31.00 [20.00,69.00]	23.50 [18.00, 38.75]	0.012^*^
Indwelling catheter	107 (76.43)	33 (75.00)	74 (77.08)	0.956
Removal of catheter 7d within SM bacteremia onset	10 (9.35)	2 (6.07)	8 (10.81)	0.721
Temperature ≥39°C	47 (33.57)	19 (43.18)	28 (29.17)	0.151
First febrile episode within past 14 days	24 (17.14)	7 (15.91)	17 (17.71)	0.984
SOFA score (median, [IQR])	4.00 [4.00, 5.00]	6.00 [5.00, 7.00]	4.00 [4.00, 5.00]	<0.001^*^
Underlying hematological disease				
Acute myeloid leukemia	58 (41.43)	19 (43.18)	39 (40.62)	0.920
Aplastic anemia	35 (25.00)	13 (29.55)	22 (22.92)	0.528
Acute lymphoblastic leukemia	24 (17.14)	3 (6.82)	21 (21.88)	***0.051***
Myelodysplastic syndrome	15 (10.71)	5 (11.36)	10 (10.42)	1
Chronic myelocytic leukemia	3 (2.14)	1 (2.27)	2 (2.08)	1
Lymphoma	2 (1.43)	1 (2.27)	1 (1.04)	1
Chronic lymphocytic leukemia	1 (0.71)	1 (2.27)	0 (0.00)	0.314
Multiple myeloma	1 (0.71)	1 (2.27)	0 (0.00)	0.314
Other	1 (0.71)	0 (0.00)	1 (1.04)	1
Treatment of hematological diseases				
Chimeric antigen receptor T-cell therapy	3 (2.14)	1 (2.27)	2 (2.08)	1
Hematopoietic stem cell transplantation	22 (15.71)	8 (18.18)	14 (14.58)	0.770
Chemotherapy	102 (72.86)	29 (65.91)	73 (76.04)	0.295
Glucocorticoid use	79 (56.43)	26 (59.09)	53 (55.21)	0.805
Previous SM infection at other sites	58 (41.43)	23 (52.27)	35 (36.46)	0.114
Polymicrobial bacteremia	10 (7.14)	5 (11.36)	5 (5.21)	0.287
Source of bacteremia				
Primary	81 (57.86)	22 (50.00)	59 (61.46)	0.276
Lung	20 (14.29)	11 (25.00)	9 (9.38)	0.028^*^
Oropharyngeal	29 (20.71)	9 (20.45)	20 (20.83)	1
Intestinal	2 (1.43)	1 (2.27)	1 (1.04)	0.531
Perianal	6 (4.29)	1 (2.27)	5 (5.21)	0.665
Skin and soft tissue	2 (1.43)	0 (0.00)	2 (2.08)	1
Co-infections				
Pulmonary infection	105 (75.00)	39 (88.64)	66 (68.75)	0.021^*^
Skin and soft tissue infection	41 (29.29)	10 (22.73)	31 (32.29)	0.340
Perianal infection	27 (19.29)	6 (13.64)	21 (21.88)	0.360
Gastroenteritis	15 (10.71)	3 (6.82)	12 (12.50)	0.390
Oral mucositis/pharyngitis	47 (33.57)	14 (31.82)	33 (34.38)	0.917
Hypoalbuminemia	106 (75.71)	39 (88.64)	67 (69.79)	0.028^*^
Bloody sputum or hemoptysis	21 (15.00)	13 (29.55)	8 (8.33)	0.003^*^
Septic shock within 24 hours	8 (5.71)	7 (15.91)	1 (1.04)	0.001^*^
Respiratory failure within 24 hours	10 (7.14)	10 (22.73)	0 (0.00)	<0.001^*^
Prolonged neutropenia (≥14 days)	97 (69.29)	36 (81.82)	61 (63.54)	0.048^*^

^*a*^ Data are presented as median (IQR), mean (SD), or n (%). A *P* value in italics and bold means <0.1, followed by * means <0.05.

^*b*^ CAR-T, Chimeric antigen receptor T-cell therapy; HSCT, Hematopoietic stem cell transplantation; SM, *S. maltophilia*; SOFA, Sequential organ failure assessment.

In this cohort of 140 patients, the median length of hospital stay before the occurrence of SM bacteremia was 25 days (IQR: 18.00–48.50), significantly longer in non-survivors than in survivors (31.00 [IQR: 20.00–69.00] vs 23.50 [IQR: 18.00–38.75] days, *P* = 0.012). All patients developed fever at the onset of bacteremia, with the majority (97.14%, 136/140) presenting with febrile neutropenia, and 116 cases (82.86%) experiencing persistent or recurrent fever.

In the non-survivor group, patients with neutropenia lasting ≥14 days were more common compared with the survivor group (81.82% vs 63.53%, *P*=0.048). Non-survivors were more likely to present with pulmonary infection (88.64% vs 68.75%, *P* = 0.021) and hypoalbuminemia (88.64% vs 69.79%, *P* = 0.028). The median SOFA score of non-survivors was also higher than that of survivors (6.00 [IQR: 5.00, 7.00] vs 4.00 [IQR: 4.00, 5.00], *P* < 0.001). Septic shock occurred in eight patients, and 10 patients experienced respiratory failure within 24 h of SM bacteremia onset. Only 59 cases (42.14%) had a definite source of bacteremia, with the oropharynx (49.15%) and lungs (33.90%) being the most common sources.

### Antibiotic treatments

The antibiotic treatments are detailed in Table 2; Table S1. In the previous 1 month, all patients had been on broad-spectrum antibiotics for a median duration of 17 days (IQR: 11.00–23.25), and 96.43% (135/140) had received carbapenem for a median duration of 9 days (IQR: 7.00–14.00). Notably, 69.29% (97/140) developed breakthrough bacteremia during carbapenem therapy for more than 48 h. Over half of the patients (53.37%) had received more than three kinds of antibiotics in the previous 1 month, with the most prevalent antibiotic combination therapy involving carbapenems and anti-methicillin-resistant staphylococcus aureus (anti-MRSA) drugs (51.43%) (Table S1). Of note, patients in the non-survivor group had been exposed to broad-spectrum antibiotics for a significantly longer duration compared with the survivors (22 days vs 16 days, *P* < 0.001). The use of fluoroquinolones (27.27% vs 9.38%, *P* = 0.013) and tigecycline (31.82% vs 6.25%, *P* < 0.001) was more frequent among non-survivors.

**TABLE 2:** Comparison of antibiotic usage before and after bacteremia between non-survivors and survivors*^a^*^,^*^b^*

	Total *n* = 140	Non-survivor *n* = 44	Survivor *n* = 96	*P* value
Antibiotics used in the previous 1 month				
Carbapenems	135 (96.43)	41 (93.18)	94 (97.92)	0.179
anti-MRSA	74 (52.86)	28 (63.64)	46 (47.92)	0.122
Aminoglycosides	14 (10.00)	5 (11.36)	9 (9.38)	0.765
Fluoroquinolones	21 (15.00)	12 (27.27)	9 (9.38)	0.013^*^
BLBLIs	62 (44.29)	25 (56.82)	37 (38.54)	***0.066***
Cephalosporins	37 (26.43)	14 (31.82)	23 (23.96)	0.440
Tigecycline	20 (14.29)	14 (31.82)	6 (6.25)	<0.001^*^
Prior 1-month carbapenem duration, days, (median, [IQR])	9.00 [7.00, 14.00]	11.00 [7.00, 15.00]	9.00 [6.75, 13.00]	***0.092***
Breakthrough infection during carbapenem therapy	97 (69.29)	26 (59.09)	71 (73.96)	0.116
Antibiotics ≥ 3 kinds	75 (53.57)	28 (63.64)	47 (48.96)	0.152
Duration of broad-spectrum antibiotics use in preceding 1 month, days, (median, [IQR])	17.00 [11.00, 23.25]	22.00 [14.75, 30.00]	16.00 [9.75, 21.00]	<0.001^*^
Broad-spectrum antibiotics use ≥21 days	52 (37.14)	25 (56.82)	27 (28.12)	0.002^*^
Antifungal drugs	137 (97.86)	44 (100.00)	93 (96.88)	0.552
Antibiotic therapy after SM bacteremia				
Fluoroquinolones	89 (63.57)	28 (63.64)	61 (63.54)	1.000
BLBLIs	98 (70.00)	26 (59.09)	72 (75.00)	***0.088***
Cefoperazone/sulbactam	75 (53.57)	18 (40.91)	57 (59.38)	***0.064***
Piperacillin/tazobactam	30 (21.43)	7 (15.91)	23 (23.96)	0.392
Ceftazidime/avibactam	11 (7.86)	6 (13.64)	5 (5.21)	0.100
Cefoperazone/tazobactam	2 (1.43)	0 (0.00)	2 (2.08)	1.000
Cephalosporins	28 (20.00)	5 (11.36)	23 (23.96)	0.133
Tigecycline	104 (74.29)	35 (79.55)	69 (71.88)	0.450
TMP/SMX	80 (57.14)	19 (43.18)	61 (63.54)	0.038^*^
Appropriate therapy started within 24 h	34 (24.29)	12 (27.27)	22 (22.92)	0.730
Monotherapy	23 (67.65)	9 (75.00)	14 (63.64)	
Combination therapy	11 (32.35)	3 (25.00)	8 (36.36)	
Appropriate therapy started within 48 h	83 (59.29)	27 (61.36)	56 (58.33)	0.878
Monotherapy	44 (53.01)	16 (59.26)	28 (50.00)	
Combination therapy	39 (46.99)	11 (40.74)	28 (50.00)	

^*a*^ Data are presented as median (IQR), mean (SD), or n (%). A *P* value in italics and bold means <0.1, followed by * means <0.05.

^*b*^ anti-MRSA, anti-methicillin-resistant Staphylococcus aureus; BLBLIs, β-lactam-β-lactamase inhibitor combinations; SM, *S. maltophilia*; TMP/SMX: trimethoprim-sulfamethoxazole.

Following the onset of SM bacteremia, over half of the patients (59.29%) received appropriate empirical therapy within 48 h. Among them, 44 (53.01%) received monotherapy, and 39 (46.99%) received combination therapy. Additionally, tigecycline was the most frequently prescribed antibiotic (71/140, 50.71%) within 48 h of SM bacteremia onset, and non-survivors had a slightly higher usage rate of tigecycline compared with the survivors (63.64% vs 44.79%, *P* = 0.059). Notably, survivors were more likely to receive TMP/SMX (63.54% vs 43.18%, *P* = 0.040) and cefoperazone/sulbactam (CSL) (59.38% vs 40.91%, *P* = 0.064). However, the use of other β-lactam-β-lactamase inhibitor combinations (piperacillin/tazobactam, ceftazidime/avibactam, and cefoperazone/tazobactam) or fluoroquinolones subsequent to SM bacteremia did not show a significant difference between the two groups.

### Risk factors associated with 28-day mortality

Univariate analysis was performed to identify potential factors associated with 28-day mortality (Tables 1 and 2). Since the SOFA score includes parameters related to septic shock and respiratory failure, to avoid redundancy and collinearity, the latter two factors were excluded. Consequently, 15 candidate variables were included in the multivariate logistic analysis using stepwise selection, and four independent risk factors were identified: SOFA score ≥5 (OR = 10.55, 95% CI 3.49–31.91, *P* < 0.001), pulmonary infection (OR = 4.05, 95% CI 1.01–16.27, *P* = 0.049), tigecycline use in the previous 30 days (OR = 9.52, 95% CI 2.40–37.75, *P* = 0.001) and age ≥60 years (OR = 4.94, 95% CI 1.43–17.11, *P* = 0.012) (Table 3).

**TABLE 3:** Multivariate analysis, risk score, and multicollinearity assessment of 28-day mortality in adult hematological patients with SM bacteremia*^a^*^,^*^b^*

	Univariate analysis		Multivariate analysis		
	OR (95% CI)	*P* value	OR (95% CI)	*P* value	VIF
Age ≥ 60 years	2.58 (1.02–6.51)	0.046^*^	4.94 (1.43–17.11)	0.012^*^	1.138
Length of hospital stay before SM bacteremia ≥30 days	2.29 (1.11–4.74)	0.026^*^			
Hypoalbuminemia	3.38 (1.21–9.44)	0.020^*^			
SOFA score ≥5	11.63 (4.65–29.06)	<0.001^*^	10.55 (3.49–31.91)	<0.001^*^	1.127
Acute lymphoblastic leukemia	0.26 (0.07–0.93)	0.038^*^			
Pulmonary infection	3.55 (1.27–9.89)	0.016^*^	4.05 (1.01–16.27)	0.049^*^	1.094
Source of bacteremia (lung)	3.22 (1.22–8.48)	0.018^*^			
Bloody sputum or hemoptysis	4.61 (1.75–12.18)	0.002^*^	3.3 (0.89–12.22)	***0.073***	1.070
Prolonged neutropenia before SM bacteremia	2.58 (1.08–6.17)	0.033^*^	2.68 (0.80–8.99)	0.110	1.069
Fluoroquinolones used in the preceding 1 month	3.62 (1.40–9.42)	0.008^*^			
BLBLIs use in preceding 1 month	2.10 (1.02–4.33)	0.045^*^			
Tigecycline use in preceding 1 month	7.00 (2.47–19.84)	<0.001^*^	9.52 (2.40–37.75)	0.001^*^	1.088
Broad-spectrum antibiotics use ≥21 days	3.36 (1.60–7.08)	0.001^*^			
CSL use after SM bacteremia	0.47 (0.23–0.98)	0.044^*^	0.47 (0.18–1.27)	0.138	1.021
TMP/SMX use after SM bacteremia	0.44 (0.21–0.90)	0.025^*^	0.46 (0.16–1.29)	0.140	1.100

^*a*^ Data are presented as median (IQR), mean (SD), or n (%). A *P* value in italics and bold means <0.1, followed by * means <0.05.

^*b*^ BLBLIs, β-lactam-β-lactamase inhibitor combinations; CI, confidence interval; CSL, cefoperazone/sulbactam; OR, odds ratio; SOFA, Sequential Organ Failure Assessment; SM, S. maltophilia; TMP/SMX: trimethoprim-sulfamethoxazole; VIF: variance inflation factor.

### Risk stratification and antibiotic treatment on 28-day mortality

**Fig 1:**
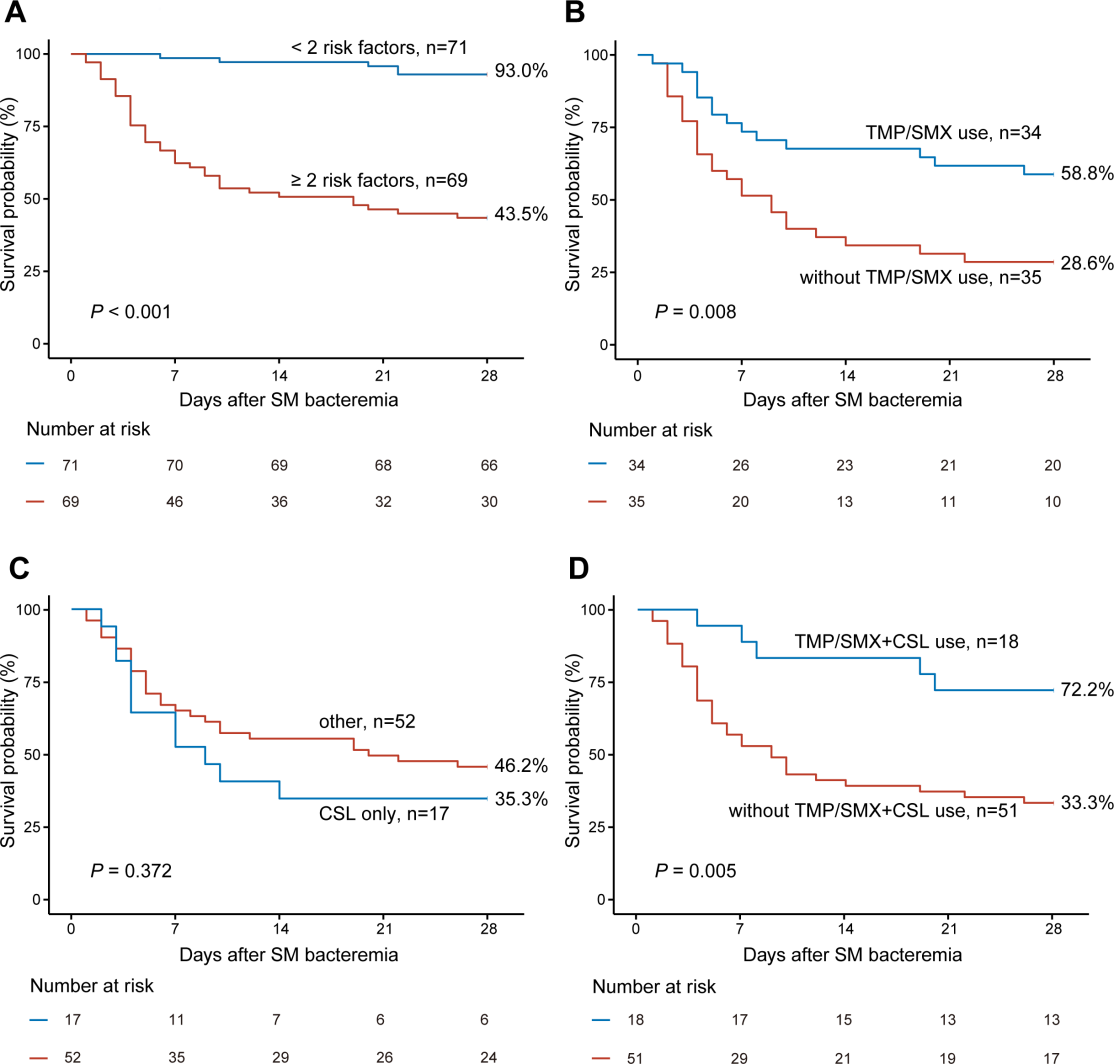
Kaplan-Meier curves of 28-day overall survival (OS) in adult hematological patients with SM bacteremia.

When comparing the 28-day survival curves of patients with different numbers of risk factors, those with ≥2 risk factors were stratified into the high-risk group, exhibiting significantly higher 28-day mortality compared with the low-risk group (56.52% vs 7.04%, *P*<0.001) (Fig. 1A). Further evaluation of the impact of antibiotic treatment on different risk populations revealed that high-risk group patients who received TMP/SMX (*P* = 0.008) or TMP/SMX combined with CSL (*P* = 0.005) showed a favorable prognosis (Fig. 1B and D). However, the use of CSL alone (*P* = 0.372) or TMP/SMX combined with levofloxacin (LVX) (*P* = 0.684) (Fig. S1) was not associated with prognosis.

### Antimicrobial susceptibility

**Fig 2:**
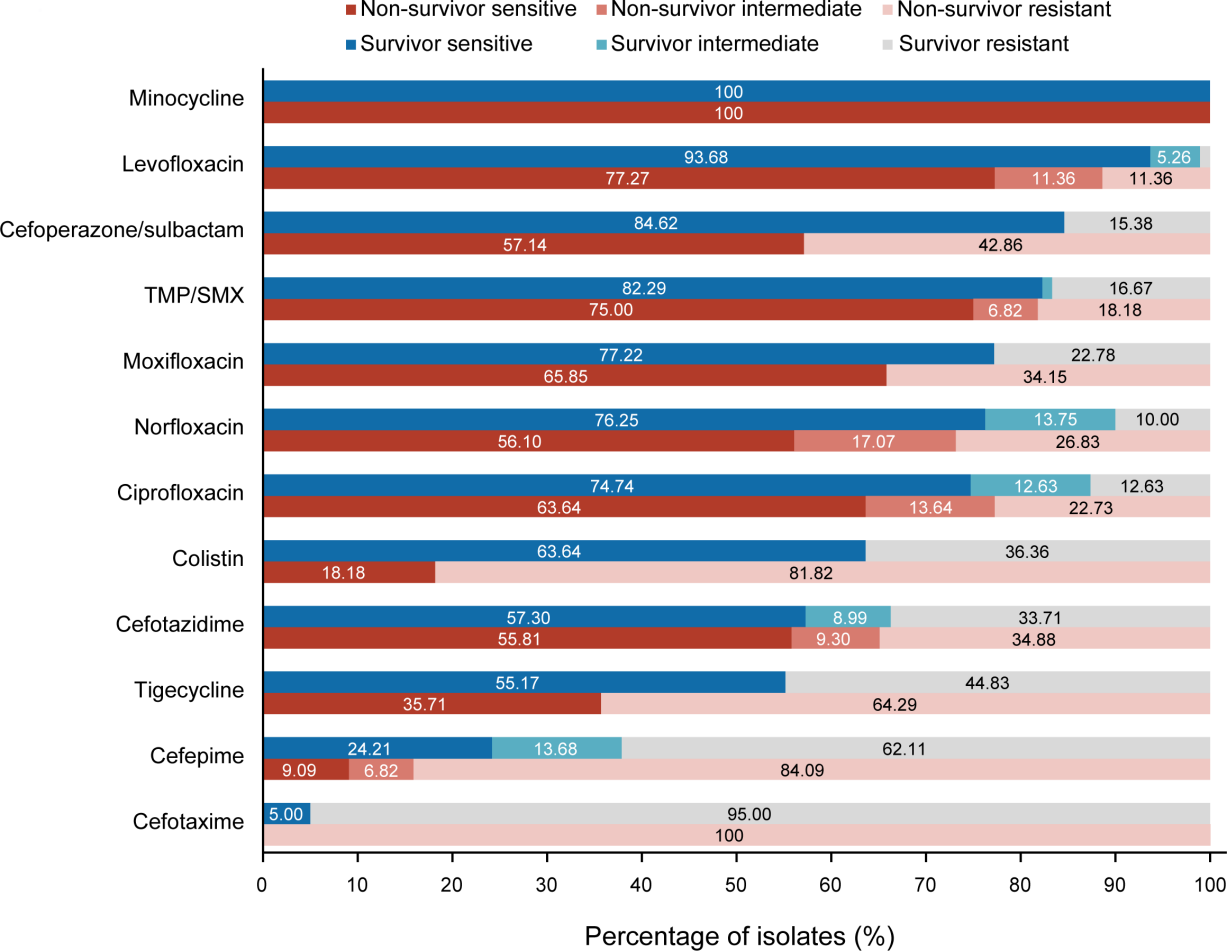
Antibiotic susceptibility of SM blood isolates to 12 antibiotics stratified by clinical outcomes.

As shown in Table S2, minocycline exhibited the highest antimicrobial activity against the isolated strains (100.00%), followed by LVX (88.49%), TMP/SMX (80.00%), and CSL (75.00%). The survivor group showed higher sensitivity to all of these antibiotics (Fig. 2).

## DISCUSSION

This single-center study is the largest case series of SM bacteremia in adult hematological patients to date. We observed a 28-day mortality rate of 31.43%, consistent with the high mortality rates reported in previous studies (7, 12, 16–18, 20). Independent adverse prognostic factors identified included higher SOFA scores, advanced age, pulmonary infection, and recent tigecycline exposure. Our findings indicated that treatment with TMP/SMX or TMP/SMX combined with CSL was effective in patients with two or more risk factors.

In this study, most patients exhibited prolonged hospitalization, previous broad-spectrum antibiotics use, particularly carbapenems, and unresolved neutropenia before bacteremia. These characteristics aligned with established high-risk factors for SM bacteremia in hematological patients (19, 24), and prior carbapenem use was the most frequently reported predisposing factor (4, 5, 19, 24, 25). A case-control study by Lee et al. (26) also identified SM as the most common pathogen in breakthrough Gram-negative bacteremia during carbapenem treatment. Therefore, clinicians should suspect the potential development of SM bacteremia in long-term hospitalized patients with hematological diseases who exhibit persistent symptoms such as chills and fever during carbapenem therapy.

In patients with SM bacteremia, prior studies have established that the severity of medical conditions (25, 27) and advanced age are significant risk factors for mortality (8, 11, 16). Our findings further highlighted that a SOFA score ≥5 and age ≥60 years were independent risk factors for 28-day mortality. Hence, it is essential to exercise caution and adopt a proactive approach in the management of critically ill and elderly patients.

Pulmonary infection was a prevalent concern among hematological patients and a well-established risk factor for both SM bacteremia and subsequent mortality (17, 28), which was further substantiated in our study. Notably, pulmonary infection served as a common source of SM bacteremia (19, 29), and our data showed that one-fifth (21/105) of patients with pulmonary infection developed bloody sputum or hemoptysis within 24 h of SM bacteremia onset. These symptoms may result from the extracellular protease StmPr1 secreted by SM, causing tissue and vascular damage and ultimately leading to fatal hemorrhagic pneumonia (18, 20, 30, 31). A meta-analysis by Huang et al. (32) revealed rapid progression of SM bacteremia with hemorrhagic pneumonia in hematological malignancies, with 7-day and 30-day mortality rates of 85.9% (55/64) and 90.1% (82/91), respectively. Unfortunately, effective treatments for hemorrhagic pneumonia remain elusive. Therefore, although hemoptysis and bloody sputum did not reach statistical significance in our multivariable analysis, possibly due to limited sample size or multifaceted etiology of these symptoms, we emphasize the importance of promptly addressing clinical signs such as cough, sputum, and hemoptysis. Providing respiratory support, empirical antibiotics and platelet transfusions remain crucial for improving patient outcomes.

A novel finding in our study was the predictive value of prior tigecycline use for 28-day mortality. Tigecycline is commonly employed as a last-line treatment for polymicrobial infections caused by multidrug-resistant Gram-positive and -negative pathogens, particularly those resistant to carbapenems (33). Consequently, individuals previously administered tigecycline tend to exhibit complicated, difficult-to-treat infections (34). Moreover, prior tigecycline exposure can induce mutational resistance in SM through mutations in the SmeDEF efflux pump negative regulator *smeT* and alterations in ribosome biogenesis proteins (35). In our study, 20 patients had received tigecycline for over 48 h in the previous 30 days, and only two of them showed blood cultures sensitive to tigecycline. However, tigecycline was frequently prescribed empirically at our center after the onset of SM bacteremia, with a slightly higher proportion of patients in the deceased group receiving it. These findings indicated that prior tigecycline use increased the likelihood of antibiotic resistance, compromising its efficacy when re-administered following SM bacteremia. Additionally, given tigecycline’s relatively low serum concentration for treating bacteremia (36), we recommend against its re-administration in patients with a high suspicion of SM bacteremia who have utilized it in the past month.

To facilitate the identification of high-risk patients for mortality, we categorized individuals with ≥2 aforementioned risk factors into the high-risk group. We further revealed that the use of TMP/SMX or TMP/SMX combined with CSL was associated with survival benefits among high-risk patients, providing additional support for current research findings. Existing studies recommend a combination therapy for treating SM infections in hematological patients, typically involving TMP/SMX along with other active antibiotics like fluoroquinolones, minocycline, or cefiderocol (2, 13, 19, 37–40). Although cephalosporins generally exhibit limited activity against SM, β-lactamase inhibitors can suppress the L2 cephalosporinase produced by SM (15, 41), and sulbactam can enhance the activity of cefoperazone against SM (42). Thus, CSL can be included in combination therapy as a viable option for SM infection (16, 43). Additionally, CSL has been observed to inhibit SM biofilm formation and reduce biofilm colony density (44). Several studies have also reported that CSL demonstrates relatively high activity against SM. A nosocomial pathogens resistance surveillance project in China found that SM had an average sensitivity of over 85% to CSL from 1996 to 2001 (45). Similarly, Fu et al. (46) reported a sensitivity rate of 82.05% (64/78). Another study focusing on hematological patients observed a sensitivity rate of 66.7% among survivors (16). According to data from the 2023 China Antimicrobial Surveillance Network (CHINET), the resistance rate of 12,190 SM isolates to CSL was 27.6% (47). These findings suggest that CSL is a valuable option for treating SM infections in China.

In addition, TMP/SMX alone acts as a bacteriostatic agent against SM, but its combinations with other antibiotics like cephalosporins have proven more effective, even when those agents are inactive alone or only intermediately susceptible based on minimum inhibitory concentrations (48, 49). The improved outcomes observed with the TMP/SMX and CSL combination in our study may be attributed to their synergistic effect (16, 46). Moreover, carbapenems, β-lactam-β-lactamase inhibitor combinations, and fourth-generation cephalosporins are commonly used in empirical antimicrobial therapy. In hematological patients with febrile neutropenia, who often have a history of carbapenem use or infections caused by carbapenem-resistant bacteria (such as carbapenem-resistant Enterobacterales, carbapenem-resistant *Acinetobacter baumannii*, or non-fermenters) (24), treatment options for empirical therapy are limited, and mortality rates are high. Therefore, careful selection of empirical antibiotic therapy is crucial. CSL, with its broad antimicrobial spectrum and relatively high activity against SM, presents a valuable option for empirical treatment in cases suspected of SM infections (50). In summary, although prompt initiation of TMP/SMX is crucial for treating SM infections (40), mortality in hematological patients with SM bacteremia remains high, and resistance to TMP/SMX is increasing annually (51–53). The combination of TMP/SMX and CSL may present a potentially effective treatment option. Further clinical and experimental studies are warranted to validate these findings and explore the underlying mechanisms.

The combination of TMP/SMX with LVX is recommended by the Infectious Diseases Society of America guidance (39). However, Araoka et al. (54) found no additional benefit from this combination compared with TMP/SMX monotherapy, and *in vitro* studies showed limited synergistic effects, with some strains even exhibiting antagonism. Our findings also do not support the use of TMP/SMX and fluoroquinolone together for SM bacteremia, possibly due to resistance development during treatment (55). Fluoroquinolone resistance increases with cumulative use (29), with 19% to 27% of patients developing resistant strains by the end of therapy (56, 57). Moreover, the SmeDEF efflux pump, a key driver of fluoroquinolone resistance, also contributes to TMP/SMX resistance, potentially leading to cross-selection of resistance between these drugs (58). Further studies are still necessary to comprehensively assess the clinical efficacy of TMP/SMX and fluoroquinolone combination therapy for SM bacteremia and develop strategies to prevent resistance.

The study provides a comprehensive overview of SM bacteremia in hematological patients, a population often underrepresented in research. To our knowledge, it represents the largest case series of SM bacteremia in this population. By analyzing clinical characteristics and mortality risk factors, this study helps clinicians identify susceptible and high-risk patients. Our findings on antibiotic usage also provide valuable insights into clinical management. Specifically, the efficacy of TMP/SMX and its combination with CSL, along with the adverse prognostic impact of prior tigecycline use, offer important guidance for improving treatment strategies in high-risk groups.

Several limitations of our study should be acknowledged. First, this study is a single-center retrospective study, and the generalizability of the results may be limited by differences in treatment protocols and the epidemiology of SM across different institutions. Second, factors such as the duration required for complete clearance of SM from blood cultures for each patient and the potential occurrence of recurrent bacteremia were not considered in the analysis, which could be explored in future studies.

In summary, for hematological patients with prolonged hospitalization and unresolved neutropenia, the presence of recurrent or persistent fever during carbapenem therapy should raise a suspicion of SM bacteremia. Independent risk factors for 28-day mortality include high SOFA scores, advanced age, pulmonary infection, and recent tigecycline exposure. Patients with two or more of these risk factors face a significantly higher risk of poor outcomes. TMP/SMX is an effective treatment, although its resistance rates are relatively high. Combination therapy with TMP/SMX and CSL may offer additional benefits for high-risk patients.

## Data Availability

The data sets used or analyzed during the current study are available from the corresponding author on reasonable request.

## SUPPLEMENTAL MATERIAL

Figure S1 spectrum.01011-24-s0001.tif Kaplan-Meier curves of 28-day overall survival (OS) in patients with {greater than or equal to}2 risk factors.

Tables S1 and S2; Figure S1 legend spectrum.01011-24-s0002.docx Table S1: Detailed antibiotic usage before and after SM bacteremia.Table S2: In vitro antibiotic susceptibility of SM blood isolates.

ASM does not own the copyrights to Supplemental Material that may be linked to, or accessed through, an article. The authors have granted ASM a non-exclusive, world-wide license to publish the Supplemental Material files. Please contact the corresponding author directly for reuse.
