# Supplementary figures and images for: Stenotrophomonas maltophilia bacteremia in adult patients with hematological diseases: clinical characteristics and risk factors for 28-day mortality
Source: Microbiol Spectr. 2024 Nov 29;13(1):e01011-24. doi: 10.1128/spectrum.01011-24 (PMC11705889; doi:10.1128/spectrum.01011-24)

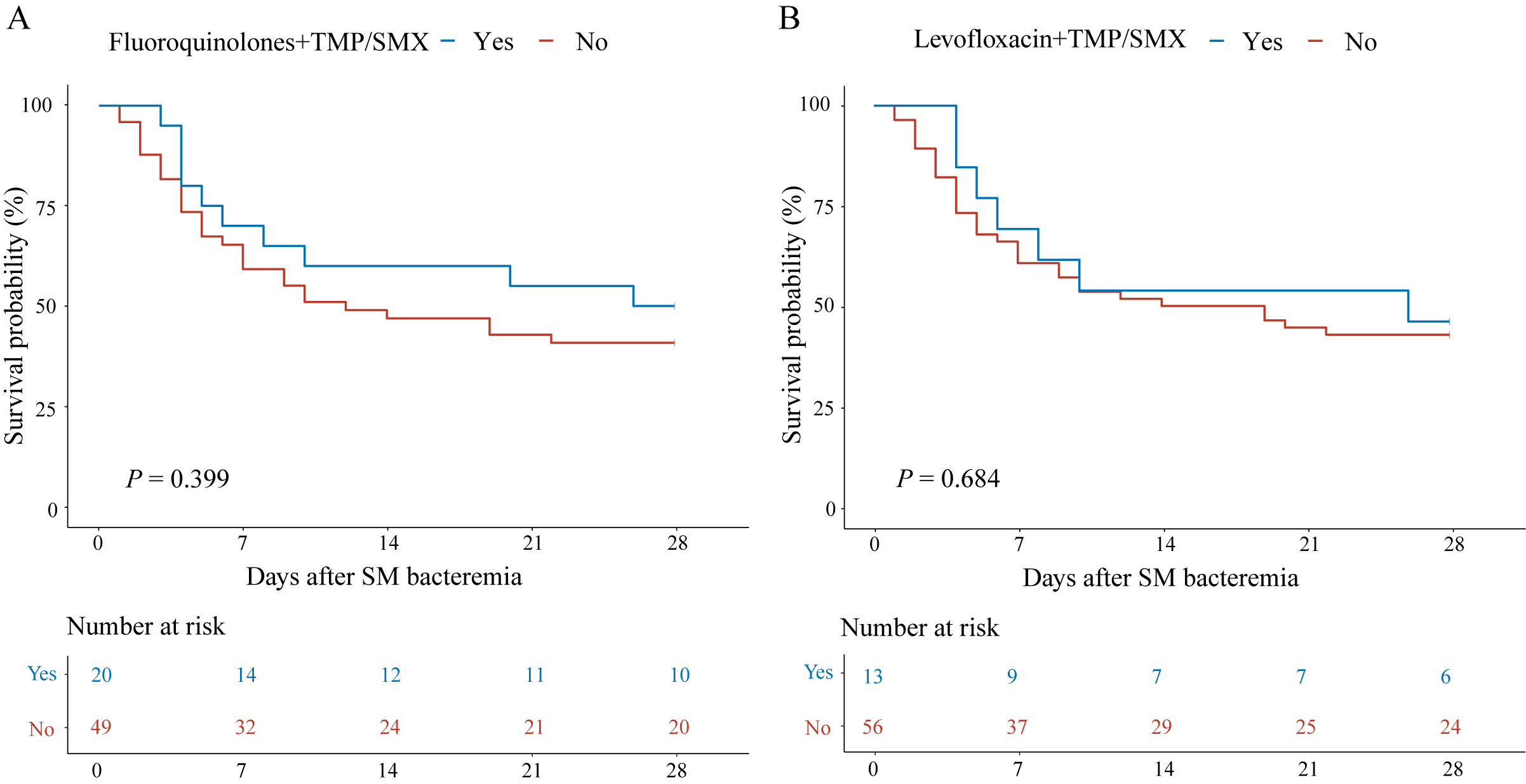

Supplement: Figure S1 — Kaplan-Meier curves of 28-day overall survival (OS) in patients with {greater than or equal to}2 risk factors. [file spectrum.01011-24-s0001.tif]
