# Supplementary material for: Stenotrophomonas maltophilia bacteremia in adult patients with hematological diseases: clinical characteristics and risk factors for 28-day mortality
Source: Microbiol Spectr. 2024 Nov 29;13(1):e01011-24. doi: 10.1128/spectrum.01011-24 (PMC11705889; doi:10.1128/spectrum.01011-24)
Supplement: Tables S1 and S2; Figure S1 legend — Table S1: Detailed antibiotic usage before and after SM bacteremia.Table S2: In vitro antibiotic susceptibility of SM blood isolates. [file spectrum.01011-24-s0002.docx]

**Supplementary Table1**: Detailed antibiotic usage before and after SM bacteremia.

|  | | Total  n=140 | Non-survivor  n=44 | Survivor  n=96 | | *P* value |  |
| --- | --- | --- | --- | --- | --- | --- | --- |
| Antibiotics used in the previous 1 month | |  |  |  | |  |  |
| Carbapenem + anti MRSA based regimes | | 72 (51.43) | 27 (61.36) | 45 (46.88) | | 0.159 |  |
| Carbapenem + anti MRSA + BLBLIs based regimes | | 36 (25.71) | 15 (34.09) | 21 (21.88) | | 0.185 |  |
| Antibiotic therapy after SM bacteremia | |  |  |  | |  |  |
| Empirical using CSL (within 48h) | | 56 (40.00) | 13 (29.55) | 43 (44.79) | | 0.128 |  |
| Empirical using fluoroquinolone (within 48h) | 50 (35.71) | | 16 (36.36) | 34 (35.42) | 1 | | |
| Empirical using tigecycline (within 48h) | 71 (50.71) | | 28 (63.64) | 43 (44.79) | ***0.059*** | | |
| Empirical using TMP/SMX (within 48h) | 32 (22.86) | | 9 (20.45) | 23 (23.96) | 0.809 | | |
| Note: Data are presented as n (%). A *P* value in italics and bold means <0.1, followed by * means <0.05.  *Abbreviations*: anti-MRSA, anti-methicillin-resistant staphylococcus aureus; BLBLIs, β-lactam-β-lactamase inhibitor combinations; CSL, cefoperazone/sulbactam; SM, *S. maltophilia*; TMP/SMX: trimethoprim-sulfamethoxazole. | | | | | | | |

**Supplementary Table 2:** In vitro antibiotic susceptibility of SM blood isolates.

| Antibiotics | Total | Non-survivors | | | Survivors | | |
| --- | --- | --- | --- | --- | --- | --- | --- |
|  | **Sensitive** | **Sensitive** | **Intermediate** | **Resistant** | **Sensitive** | **Intermediate** | **Resistant** |
| Minocycline | 35/35 (100.00) | 12/12 (100.00) | 0.00 | 0.00 | 23/23 (100.00) | 0.00 | 0.00 |
| Levofloxacin | 123/139 (88.49) | 34/44 (77.27) | 5/44 (11.36) | 5/44 (11.36) | 89/95 (93.68) | 5/95 (5.26) | 1/95 (1.05) |
| CSL | 30/40 (75.00) | 8/14 (57.14) | 0.00 | 6/14 (42.86) | 22/26 (84.62) | 0.00 | 4/26 (15.38) |
| TMP/SMX | 112/140 (80.00) | 33/44 (75.00) | 3/44 (6.82) | 8/44 (18.18) | 79/96 (82.29) | 1/96 (1.04) | 16/96 (16.67) |
| Moxifloxacin | 88/120 (73.33) | 27/41 (65.85) | 0.00 | 14/41 (34.15) | 61/79 (77.22) | 0.00 | 18/79 (22.78) |
| Norfloxacin | 84/121 (69.42) | 23/41 (56.10) | 7/41 (17.07) | 11/41 (26.83) | 61/80 (76.25) | 11/80 (13.75) | 8/80 (10.00) |
| Ciprofloxacin | 99/139 (71.22) | 28/44 (63.64) | 6/44 (13.64) | 10/44 (22.73) | 71/95 (74.74) | 12/95 (12.63) | 12/95 (12.63) |
| Colistin | 16/33 (48.48) | 2/11 (18.18) | 0.00 | 9/11 (81.82) | 14/22 (63.64) | 0.00 | 8/22 (36.36) |
| Cefotazidime | 75/132 (56.82) | 24/43 (55.81) | 4/43 (9.30) | 15/43 (34.88) | 51/89 (57.30) | 8/89 (8.99) | 30/89 (33.71) |
| Tigecycline | 63/129 (48.84) | 15/42 (35.71) | 0.00 | 27/42 (64.29) | 48/87 (55.17) | 0.00 | 39/87 (44.83) |
| Cefepime | 27/139 (19.42) | 4/44 (9.09) | 3/44 (6.82) | 37/44 (84.09) | 23/95 (24.21) | 13/95 (13.68) | 59/95 (62.11) |
| Cefotaxime | 4/121 (3.31) | 0.00 | 0.00 | 41/41 (100.00) | 4/80 (5.00) | 0.00 | 76/80 (95.00) |
| Note: Data are presented as n (%).  *Abbreviations*: CSL, cefoperazone/sulbactam; SM, *S. maltophilia*; TMP/SMX: trimethoprim-sulfamethoxazole. | | | | | | | |

**Supplementary Figure 1:** Kaplan-Meier curves of 28-day overall survival (OS) in patients with ≥2 risk factors. **(A)** Kaplan-Meier 28-day survival curves for patients with or without TMP/SMX and fluoroquinolones treatment after SM bacteremia. **(B)** Kaplan-Meier 28-day survival curves for patients with or without TMP/SMX and levofloxacin treatment after SM bacteremia.
